# A Parameter-Agnostic Adaptive Compensation in Memristor-Based Neuromorphic Systems for Parasitic Resistance

**DOI:** 10.3390/mi17040481

**Published:** 2026-04-16

**Authors:** Texu Liu, Hanbo Ren, Peiwen Tong, Wei Wang, Qingjiang Li, Meng Xia, Yi Sun, Rongrong Cao, Bing Song, Zhiwei Li, Haijun Liu

**Affiliations:** 1 College of Electronic Science and Technology, National University of Defense Technology, Changsha 410073, China; liutexu20@nudt.edu.cn (T.L.); ren030395@163.com (H.R.); tongpeiwen20@nudt.edu.cn (P.T.); sunyi12@nudt.edu.cn (Y.S.); caorongrong@nudt.edu.cn (R.C.); songbing@nudt.edu.cn (B.S.); lizhiwei@nudt.edu.cn (Z.L.); liuhaijun@nudt.edu.cn (H.L.); 2 College of Management, Ocean University of China, Qingdao 266100, China; mxia2026@163.com

**Keywords:** neuromorphic computing, memristor array, parasitic resistance, current compensation

## Abstract

Memristor-based neuromorphic computing offers a promising pathway for efficient in-memory processing. However, the scalability and reliability of such systems are severely compromised by parasitic resistances (including line and input resistances) in crossbar arrays, which cause significant IR-drop during vector–matrix multiplication (VMM). Existing research often suffers from high computational latency or relies on the precise extraction of parasitic parameters, which is impractical and computationally expensive for large-scale integration. To overcome these limitations, we propose a Parameter-Agnostic Adaptive Compensation (PAAC) method based on a distributed linear approximation model. By analyzing the circuit characteristics, we conquered the challenge of coupling between parasitic effects and output current, deriving a simplified linear relationship that requires no prior knowledge of specific resistance values. The PAAC method involves only a single-step pre-calibration experiment to determine a global compensation factor, achieving an ultra-low computational complexity during inference. We validated the method using a comprehensive two-stage strategy: board-level hardware experiments confirmed its feasibility by reducing current distortion from 71% to 2%, while extensive large-scale HSPICE simulations verified its scalability, restoring classification accuracy from 89% to 95%. This work provides a robust, low-overhead solution that eliminates the dependency on precise parameter modeling, facilitating the realization of large-scale, high-precision neuromorphic hardware.

## 1. Introduction

**Figure 1:**
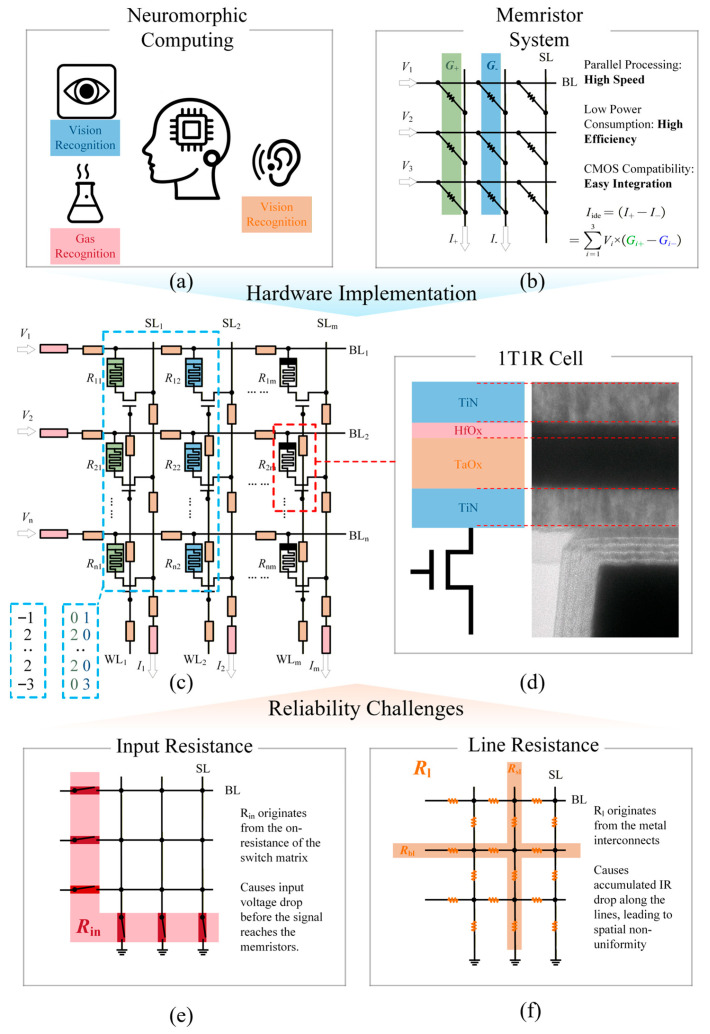
Overview of the memristor-based neuromorphic computing system and parasitic resistance models. (**a**) Conceptual illustration of neuromorphic computing tasks utilizing memristor networks. (**b**) Memristor crossbar array acting as a hardware accelerator for vector–matrix multiplication (VMM). (**c**) Equivalent circuit model of the 1T1R array highlighting parasitic resistances. (**d**) 1T1R cell structure schematic and TEM image. (**e**) Illustration of input resistance (*R*_in_). (**f**) Illustration of line resistance (*R*_l_).

Memristor-based neuromorphic computing has garnered extensive attention for breaking the von Neumann bottleneck, enabling efficient in-memory computing that aligns with the parallel processing paradigm of the human brain [1,2,3,4,5,6,7]. Memristor crossbar arrays, the core of such systems, implement vector–matrix multiplication (VMM) operations through Kirchhoff’s laws—mapping neural network inputs to row voltages and weights to memristor conductance, then extracting column currents as computation results [8,9,10,11]. As shown in Figure 1a,b, this architecture has been successfully applied in biomimetic perception (auditory, visual, tactile) and pattern classification tasks (digit, sequence, voice recognition), because of its potential for high-performance, low-power computing.

Despite these advantages, the scalability and accuracy of memristor arrays are severely limited by parasitic resistances inherent in hardware implementations. These parasitic effects are caused by two sources: input resistance (on-resistance of array switches) and line resistance (resistance of metal interconnects between memristors and switches). The voltage division effect induced by these resistances distorts the output current during VMM, introducing errors in neural network computations and degrading model performance.

Existing research on parasitic resistance compensation has focused on addressing either input resistance or line resistance in isolation, with complex computation processes that increase system latency or require precise modeling of parasitic parameters [12,13,14,15,16,17,18,19,20,21,22]. For large-scale arrays, parasitic values are difficult to quantify accurately. There remains an urgent need for a simple, universal compensation method that can mitigate the combined effects of both input and line resistance without prior knowledge of their specific values.

In this paper, we propose a Parameter-Agnostic Adaptive Compensation (PAAC) method to resolve the output current distortion caused by parasitic resistances in memristor arrays. The key contributions of this work follow: (1) Development of linear approximation compensation models for both input and line resistance, leveraging the fact that parasitic resistance values are much smaller than memristor resistance. (2) Design of the PAAC method, which requires only one pre-experiment and four multiplication operations (O(1) time complexity) to compute the compensation coefficient, avoiding the need for parasitic resistance modeling or measurement. (3) Verification of the generality and effectiveness of the PAAC method through multi-platform experiments, confirming its stability and engineering practicality in different implementation scenarios.

To validate the effectiveness of the PAAC method, we adopted a two-stage verification strategy. First, we implemented the method on a board-level memristor array system to confirm its practicality and robustness in real-world hardware environments. Subsequently, to overcome the size limitations of the hardware prototype and investigate the method’s performance in larger-scale arrays where parasitic effects are more severe, we conducted extensive HSPICE simulations on larger neural networks. Results show that the method reduces current distortion from 71% to 2% in hardware and 35% to 1% in large-scale simulation, significantly restoring neural network accuracy. Compared with state-of-the-art compensation methods, the PAAC method achieves comparable or better performance with lower complexity, making it suitable for both digital and analog circuit implementations.

The remainder of this paper is organized as follows: Section 2 analyzes the parasitic resistance effects in memristor arrays. Section 3 presents the linear compensation models for input and line resistance, followed by the detailed PAAC method. Section 4 describes the experimental verification, starting with hardware validation followed by large-scale simulation. Section 5 concludes the work and discusses future directions.

## 2. Parasitic Resistance Analysis in Memristor-Based Neuromorphic Computing

In memristor-based neuromorphic computing, the memristor array is primarily used to accelerate the inference computation process of neural networks. The core advantage of the memristor array lies in its efficient handling of vector–matrix multiplication (VMM) operations.

The input value of the neural network can be mapped to the input voltage of the memristor array, as shown in Equation (1).

(1) $$V_{\mathrm{in}}=\frac{x_{\mathrm{in}}-x_{\min}}{x_{\max}-x_{\min}}\times \left(V_{\max}-V_{\min}\right)+V_{\min}$$

where *V*_in_ is the input voltage of the memristor array and *x*_in_ represents the input values of the neural network. *V*_max_ and *V*_min_ are the maximum and minimum input voltage of the memristor array. *x*_max_ and *x*_min_ are the maximum and minimum input values of the neural network.

Then, the suitable voltage pulse parameters applied to BLs (Bit Lines) and SLs (Source Lines) can control the conductance of the 1T1R unit, which means the weights in the neural network can be mapped to the corresponding memristor conductance value. Considering that the resistance of memristors is non-negative, the array employs a differential mapping, and the mapping formula is as follows (*j*-th column):

(2) $$G_{i+}=S^{+}\times \left|\frac{a_{i}}{\left|a_{\max}\right|}\right|\times \left(G_{\max}-G_{\min}\right)+G_{\min}\;G_{i-}=S^{-}\times \left|\frac{a_{i}}{\left|a_{\max}\right|}\right|\times \left(G_{\max}-G_{\min}\right)+G_{\min}$$

(3) $$\left\{\begin{array}{l} S^{+}=0\;\mathrm{and}\;S^{-}=1,a_{i}\leq 0\\ S^{+}=1\;\mathrm{and}\;S^{-}=0,a_{i}\geq 0 \end{array}\right.$$

where *a_i_* represents the trained neural network weights. *G_i_*_+_ and *G_i_*_−_ represent the positive and negative conductance values of the *i*-th row. *a*_max_ is the maximum absolute value of the neural network weights. *G*_max_ and *G*_min_ are the maximum and minimum conductance used for computation in the memristor array, as shown in Figure 1c.

According to Kirchhoff’s laws, the ideal output current of the array (the neural network calculation result) is as follows:

(4) $$I_{\mathrm{ide}j}=\sum_{i=1}^{n}V_{i}\times \left(G_{i+}-G_{i-}\right),j\in \{1,2,\ldots ,m\}$$

A memristor array is commonly formed in a 1T1R structure to suppress leakage current, and a memristor connecting to a transistor drain forms a 1T1R unit. As shown in Figure 1d, the array transistors were fabricated using a standard 0.18 μm CMOS process, with the devices subsequently integrated in the laboratory. Transmission electron microscopy (TEM) reveals a TiN/HfO_x_/TaO_x_/TiN heterostructure with well-defined interfaces.

However, the on-resistance of the switch matrix (input resistance), as the red resistance in Figure 1e, and the line resistance of metal wires (line resistance), as the orange resistance in Figure 1f, causes distortion of the output current. To analyze the impact of the parasitic resistance in a memristor array, we conducted the simulation experiment in three aspects: the input resistance, the line resistance and the size of the memristor array. Vector–matrix multiplication is simulated on a memristor array, and the current error is defined as the ratio of the difference between the practical output current and the ideal current to the ideal current, as Equation (5).

(5) $$Current\;error=\frac{I_{\mathrm{pra}}-I_{\mathrm{ide}}}{I_{\mathrm{ide}}}$$

All simulation experiments were performed in HSPICE. For each input resistance value (from 50 Ω to 1000 Ω, 50 Ω per step), each line resistance value (from 5 Ω to 100 Ω, 5 Ω per step), and each size of memristor arrays (from 8 × 8 to 64 × 64, 8 per step), 20 different memristor arrays are generated and tested. Each memristor array consists of random input voltage (0–0.3 V) and random memristor resistance (10–100 kΩ). Considering that the input voltage and weights are unchanged during the neural network calculation, the input voltage and the parasitic resistance in the memristor array are randomly generated in a uniform distribution and remain constant during the simulation.

**Figure 2:**
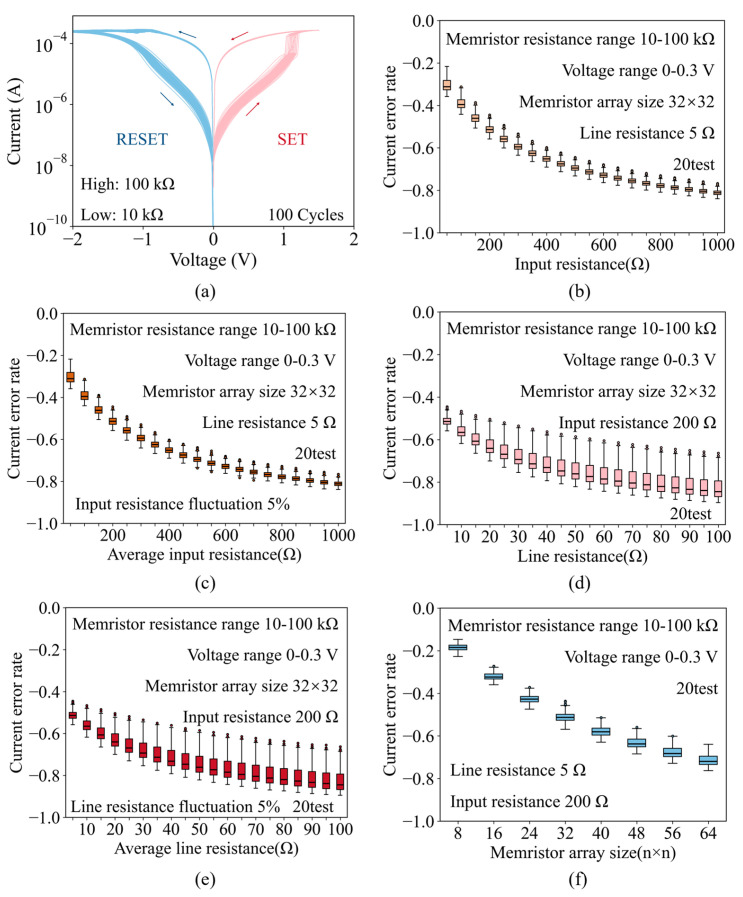
Analysis of the parasitic resistance problem. (**a**) I-V switching characteristics of the 1T1R cell. (**b**) The impact of input resistance on the output current. (**c**) The impact of input resistance ±5% fluctuation on the output current. (**d**) The impact of line resistance on the output current. (**e**) The impact of line resistance ±5% fluctuation on the output current. (**f**) The impact of the size of the 1T1R array on the output current.

Figure 2a presents the current–voltage (I-V) characteristics from 100 consecutive cycling tests, confirming the typical resistive switching behavior of the device. The low- and high-resistance states of the memristor are approximately 10 kΩ and 100 kΩ, respectively, which provide the resistance boundaries for the subsequent simulation modeling and experiments. It should be emphasized that the subsequent HSPICE and system-level simulations do not use only these two discrete states. Instead, the memristor resistances are randomly generated within the approximate range from 10 kΩ to 100 kΩ, thereby naturally covering a large number of intermediate states. Meanwhile, the 0–0.3 V input range considered in this work corresponds to the inference/read stage, during which the programmed conductance states are assumed to remain unchanged within a single computation, and no state transition is involved. Therefore, the present work focuses on parasitic resistance compensation during the inference stage under fixed programmed states, while the extension of the PAAC method to weight update dynamics and memristor plasticity is left for future study.

As shown in Figure 2b, the statistical results of the current error caused by the input resistance show that with the increase in the input resistance, the absolute value of the current error also increases. Considering that the input resistance of various lines may be different in practical situations, we introduce ± 5% fluctuation of input resistance, as shown in Figure 2c. The statistics trends are similar in Figure 2b, which means small input resistance fluctuations can be ignored. Similar to the input resistance, the statistical results of the current error caused by the line resistance show that with the increase in the line resistance, the absolute value of the current error also increases, as shown in Figure 2d. Also, small line resistance fluctuations can be ignored, as shown in Figure 2e. Finally, as shown in Figure 2f, the increase in the array size causes the number of rows for parallel computing to increase, and the absolute value of the current error also increases. Therefore, as the size of the array increases, the influence of parasitic resistance becomes worse, and the compensation requirement of the parasitic resistance effect becomes more significant. The above results indicate that parasitic resistance in the memristor array can cause serious errors in calculation output, and an effective compensation method is an urgent need in practical applications.

## 3. Parameter-Agnostic Adaptive Compensation for Parasitic Resistance

### 3.1. Linear Approximation–Compensation Model for the Input Resistance Circuit

Here, we analyze the influence of input resistance on the circuit based on Kirchhoff’s laws and propose a linear approximation model that includes input resistance, which is used for thew compensation of input resistance.

We simplified the memristor array in Figure 1c into a circuit model with fixed-input resistances. This modification aims to simplify the complex memristor array and retain the core characteristics of the input resistances. According to Ohm’s law and Kirchhoff’s laws, the ideal output current *I*_ide_ and the practical output current *I*_pra_ of the *j*-th column are expressed:

(6) $$I_{\mathrm{ide}}=\sum_{i=1}^{n}\frac{V_{i}}{R_{ij}}$$

(7) $$I_{\mathrm{pra}}=\sum_{i=1}^{n}\frac{V_{i}-R_{\mathrm{in}}\times I_{\mathrm{pra}}}{R_{ij}+R_{\mathrm{in}}}$$

where *V_i_* represents the input voltage of BL*_i_* and *R_ij_* represents the resistance of the 1T1R cell in the *i*-th row and *j*-th column. The input resistance *R*_in_ is much smaller than *R_ij_*. Expanding Equation (7), we can obtain:

(8) $$I_{\mathrm{pra}}\times \left(R_{1j}+R_{\mathrm{in}}\right)\cdots \left(R_{nj}+R_{\mathrm{in}}\right)\;=\left(V_{1}-R_{\mathrm{in}}I_{\mathrm{pra}}\right)\;\times \left[\left(R_{2j}+R_{\mathrm{in}}\right)\cdots \left(R_{nj}+R_{\mathrm{in}}\right)\right]+\cdots +\left(V_{n}-R_{\mathrm{in}}I_{\mathrm{pra}}\right)\;\times \left[\left(R_{1j}+R_{\mathrm{in}}\right)\cdots \left(R_{\left(n-1\right)j}+R_{\mathrm{in}}\right)\right]$$

We expand the polynomials on the left side of Equation (8) using binomial expansion. Considering the input resistance *R*_in_ is much smaller than the memristor resistance *R_ij_*, the contribution of the second- and higher-order terms of *R*_in_ is negligible. Therefore, only the zeroth- and first-order terms are retained, as shown in Equation (9).

(9) $$\left(R_{1j}+R_{in}\right)\cdots \left(R_{nj}+R_{in}\right)\approx R_{1j}\times \cdots \;\times R_{nj}+R_{in}\left(R_{2j}\cdots R_{nj}+\cdots +R_{1j}\cdots R_{\left(n-1\right)j}\right)$$

Equation (10) can be obtained by bringing Equation (9) into Equation (8) and simplifying it:

(10) $$\sum_{i=1}^{n}\frac{V_{i}}{R_{ij}}=I_{ide}\approx I_{com}=A\times I_{pra}+B$$

(11) $$A=\left[1+2R_{\mathrm{in}}\left(\frac{1}{R_{1j}}+\frac{1}{R_{2j}}+\cdots +\frac{1}{R_{nj}}\right)+2R_{\mathrm{in}}^{2}\left(\frac{1}{R_{1j}R_{2j}}+\frac{1}{R_{1j}R_{3j}}+\cdots +\frac{1}{R_{1j}R_{nj}}+\frac{1}{R_{2j}R_{3j}}+\cdots +\frac{1}{R_{\left(n-1\right)j}R_{nj}}\right)\right]$$

(12) $$B=-R_{\mathrm{in}}\left[\frac{V_{1}}{R_{1j}}\left(\frac{1}{R_{2j}}+\frac{1}{R_{3j}}+\cdots +\frac{1}{R_{nj}}\right)+\cdots +\frac{V_{n}}{R_{nj}}\left(\frac{1}{R_{1j}}+\frac{1}{R_{2j}}+\cdots +\frac{1}{R_{\left(n-1\right)j}}\right)\right]$$

If the compensation factors are related to the input voltage *V_i_*, the compensation factors need to be recalculated for each sample data input. This is an extremely complex and unreasonable task. For this reason, we propose a reasonable simplification. We successfully remove the parameter *V_i_* by equating the memristor resistance to a column average resistance *R*_mean_.

(13) $$B=-R_{\mathrm{in}}\frac{n-1}{R_{mean}}\times I_{ide}$$

(14) $$I_{\mathrm{ide}}\approx I_{\mathrm{com}}=\frac{A}{1+R_{\mathrm{in}}\frac{n-1}{R_{mean}}}\times I_{\mathrm{pra}}$$

Therefore, under the condition that the input resistance is much smaller than the memristor resistance, the compensation for the input resistance is approximately linear, and the factors need to be calculated only once for each model. This model provides a theoretical foundation for the subsequent experimental validation of the linear compensation for parasitic resistance.

### 3.2. Linear Approximation–Compensation Model for the Line Resistance Circuit

Similar to input resistance, we analyze the effect of line resistance in the circuit and propose a linear approximation model that includes line resistance, which is used for the compensation of line resistance.

**Figure 3:**
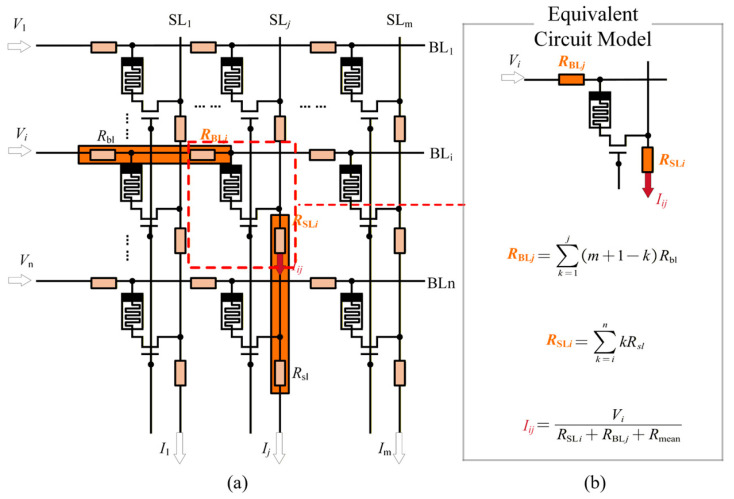
Circuit diagram of the memristor array with line resistance. (**a**) Complete circuit model. (**b**) Simplified circuit model of line resistance.

As a first step, we simplified the memristor array in Figure 3a into an equivalent circuit model to facilitate analysis. Considering that line resistance on WL does not participate in the VMM process, its impact on the output current is negligible. Consistent with the analysis of input resistance, we assume that all memristors in the array have the same average resistance *R*_mean_. This modification is intended to simplify the complex memristor array while preserving the essential properties of the line resistances. We will demonstrate that the simplified model can effectively capture the output distortion effects in Section 3.3. Since all Source Lines (SLs) are connected to the virtual ground and the line resistance is much smaller than the average resistance of the memristors, the current flowing through each memristor on the same Bit Line (BL*_i_*) is approximately equal, *I_i_*_1_ = … = *I_i_*_m_. The current flowing through *R*_bl_ in the *j*-th column is (m + 1 − *j*) × *I_i_*_1_ and the voltage drop is (m + 1 − *j*) × *I_i_*_1_*R*_bl_. Based on this analysis, the memristor array in the *j*-th column can be modeled by the equivalent circuit shown in Figure 3b. In this model, the line resistance that affects the voltage at the top node of the memristor is equivalent to a resistor *R*_BL_*_j_*.

(15) $$R_{\mathrm{BL}j}=\sum_{k=1}^{j}\left(m+1-k\right)R_{\mathrm{bl}}=\frac{\left(2m+1-j\right)j}{2}R_{\mathrm{bl}}$$

For the line resistances on the same Source Line (SL*_j_*), we assume that the input voltages are all equal, *V*_1_ = … = *V*_n_ = *V*_in_. Consistent with the analysis of input resistance, the currents flowing through the memristors in the same SL column (SL*_j_*) are approximately equal, *I*_1_*_j_* = … = *I*_n_*_j_*. The current flowing through *R*_sl_ in the *i*-th row is *i* × *I*_1_*_j_*, and the voltage drop is *i* × *I*_1_*_j_R*_sl_. Therefore, the memristor array can be further represented by the equivalent circuit at the (*i*-th row, *j*-th column) in Figure 3b. In this model, the line resistance that affects the voltage at the bottom node of the memristor is equivalent to a resistor *R*_SL_*_i_*.

(16) $$R_{\mathrm{SL}i}=\sum_{k=i}^{n}kR_{sl}=\frac{\left(n+i\right)\left(n-i+1\right)}{2}R_{sl}$$

Based on the simplified circuit in Figure 3b, the current flowing through the memristor at the (*i*-th row, *j*-th column) *I_ij_* can be represented.

(17) $$I_{ij}=\frac{V_{\mathrm{in}}}{R_{\mathrm{SL}i}+R_{\mathrm{BL}j}+R_{\mathrm{mean}}}$$

Then, the ideal output current *I*_ide_ and the practical output current *I*_pra_ of the *j*-th column are expressed:

(18) $$I_{\mathrm{ide}}=\sum_{i=1}^{n}\frac{V_{\mathrm{in}}}{R_{\mathrm{mean}}}=\frac{nV_{\mathrm{in}}}{R_{\mathrm{mean}}}$$

(19) $$I_{\mathrm{pra}}=\sum_{i=1}^{n}I_{ij}=V_{\mathrm{in}}\sum_{i=1}^{n}\frac{1}{R_{\mathrm{SL}i}+R_{\mathrm{BL}j}+R_{\mathrm{mean}}}$$

The compensation current can be obtained by bringing Equation (19) into Equation (18):

(20) $$I_{\mathrm{ide}}\approx I_{\mathrm{com}}=\frac{n}{\sum_{i=1}^{n}\frac{R_{\mathrm{mean}}}{R_{\mathrm{SL}i}+R_{\mathrm{BL}j}+R_{\mathrm{mean}}}}\times I_{\mathrm{pra}}$$

Therefore, under the condition that the line resistance is much smaller than the memristor resistance, the compensation for the line resistance is approximately linear, and the factors need to be calculated only once for each model. This model provides a theoretical foundation for the subsequent experimental validation of the linear compensation for parasitic resistance.

### 3.3. Validity and Applicability of the Linear Approximation Model

It should be emphasized that the simplifying assumptions introduced in the linear-approximation derivations of Section 3.1 and Section 3.2—such as the use of the average memristor resistance *R*_mean_, the equal-input assumption in the line resistance analysis, the approximate treatment of local node voltages, and the equivalent treatment of column-level current distortion—are adopted only to make the derivation analytically tractable and to extract the dominant parasitic-resistance-induced distortion terms. These assumptions are not intended to serve as a strict physical description of every local node voltage, branch current, or device state in a real memristor array.

Accordingly, the objective of the proposed model is not to reconstruct the exact behavior of every crosspoint in the array, but to establish a low-complexity equivalent relationship that accurately captures the dominant distortion at the column output current level, which is the final quantity directly relevant to neural network inference. From this perspective, the linear approximation should be understood as a first-order effective model for the overall output behavior, rather than a point-by-point exact circuit reconstruction.

It is also important to note that the subsequent validation does not rely on these idealized assumptions. In the HSPICE and supplementary simulations, random memristor resistances distributed from 10 kΩ to 100 kΩ and random input voltages are used, while the board-level verification is performed on a real hardware array. Therefore, the validity of the proposed method does not depend on whether all entries are physically identical or whether all inputs are exactly equal, but on whether the simplified model can still capture the dominant column-level distortion under realistic non-uniform conditions.

To further support the above discussion, additional simulations were conducted for both the input resistance case and the line resistance case. It should be emphasized that the purpose of this analysis is not to verify the internal consistency of the equivalent formulas themselves, but to evaluate the validity boundary of the linear approximation in real resistive networks. Therefore, for the input resistance case, the practical output current was obtained from an exact single-column circuit model. For the line resistance case, the practical column current was obtained from a full memristor crossbar network with distributed line resistances using nodal analysis. The resulting practical current was then substituted into the proposed linear compensation formula, and the compensated current was directly compared with the ideal current.

**Figure 4:**
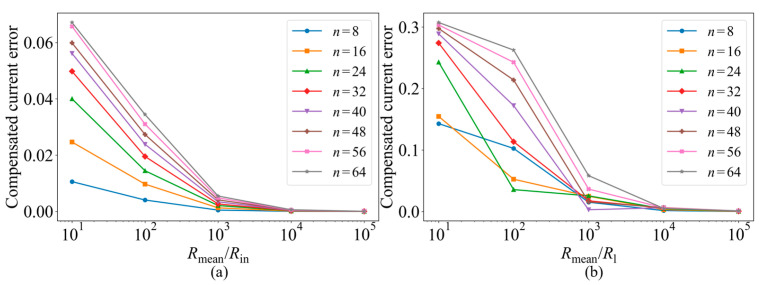
Validity boundary analysis of the linear approximation model. (**a**) Error of the compensated current relative to the ideal current versus *R*_mean_/*R*_in_ for different array sizes *n* in the input resistance case. (**b**) Error of the compensated current relative to the ideal current versus *R*_mean_/*R*_l_ for different array sizes *n* in the line-resistance case.

As shown in Figure 4, in the input resistance case, the compensation error falls below 5% when *R*_mean_/*R*_in_ ≈ 10^2^. In the line resistance case, the compensation error can be stably reduced below 5% when *R*_mean_/*R*_l_ ≈ 10^3^. Considering the practical parameter range of our experimental platform, the line resistance is typically on the order of a few ohms, the input resistance is typically on the order of tens of ohms, and the memristor resistance is typically within 10–100 kΩ [16,19]. Therefore, the practical system generally satisfies the above validity boundary conditions. This indicates that the linear equivalent model used in the subsequent experiments and board-level validation is reasonable within the practical operating range of our system.

### 3.4. Parameter-Agnostic Adaptive Compensation Method

In the previous section, the parasitic effects of input resistance and line resistance during a single operation of the memristor array were investigated, and approximate linear compensation models were proposed at the circuit level respectively. The theoretical analysis indicates that, although parasitic resistances introduce complex voltage divisions, the impact on the output current can be effectively approximated as a linear transformation. Therefore, we propose the Parameter-Agnostic Adaptive Compensation (PAAC) method, considering that when the input voltage is 0, both the ideal output current and the practical output current are 0. The relationship between the ideal output current and the practical output current can be formulated as a linear equation passing through the origin:

(21) $$I_{\mathrm{ide}}\approx I_{\mathrm{com}}=CP\times I_{\mathrm{pra}}$$

where *I*_com_ represents the compensated current and *CP* represents the compensation ratio factor between the ideal current and the practical current.

**Figure 5:**
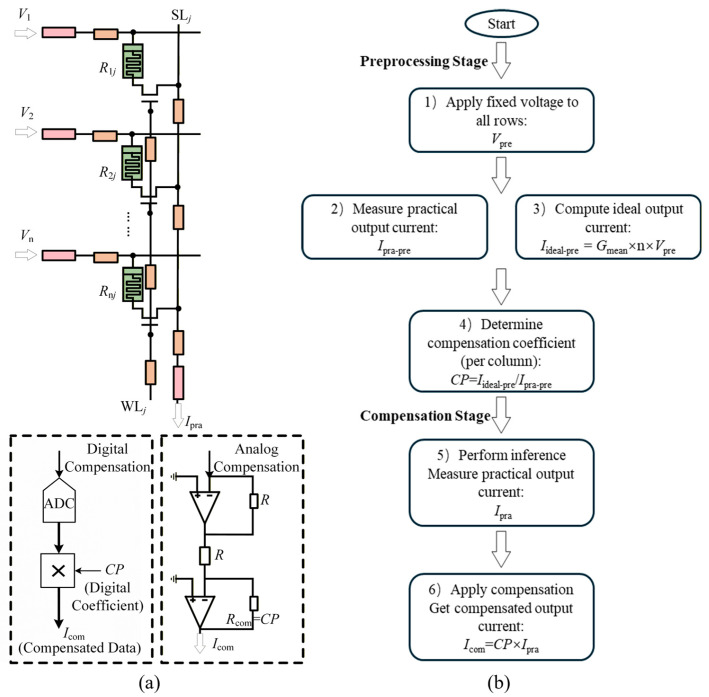
Schematic flow of the proposed Parameter-Agnostic Adaptive Compensation (PAAC) method. (**a**) Circuit diagram of the PAAC method, illustrating the hardware implementation for both analog and digital compensation paths. (**b**) Flowchart of the PAAC method, detailing the steps for pre-experiment calibration and inference.

The execution flow of the PAAC method is illustrated in Figure 5. Unlike traditional methods that require iterative solving of circuit equations or precise measurement of parasitic parameters, the PAAC method determines *CP* through a single-step pre-experiment. The specific implementation steps are as follows:

1. Pre-experiment Input: Apply a standard stability voltage *V*_pre_ (e.g., 0.3 V) to all rows of the memristor array simultaneously.
2. Measurement: Measure the practical output current *I*_pra-pre_ at the end of each column.
3. Ideal Calculation: Calculate the theoretical ideal current *I*_ide-pre_ based on the known average conductance of the column:
  (22) $$I_{\mathrm{ide}-\mathrm{pre}}=\sum_{i=1}^{n}V_{\mathrm{pre}}\times G_{i}=nV_{\mathrm{pre}}G_{\mathrm{mean}}$$
  where *G*_mean_ indicates the average conductance of the memristors on the SL.
4. Coefficient Derivation: Calculate the compensation factor *CP* for each column:
  (23) $$CP=\frac{I_{\mathrm{ide}-\mathrm{pre}}}{I_{\mathrm{pra}-\mathrm{pre}}}=\frac{nV_{\mathrm{pre}}G_{\mathrm{mean}}}{I_{\mathrm{pra}-\mathrm{pre}}}$$

It should be noted that the PAAC method does not require prior extraction of the exact input or line parasitic resistances. However, it still relies on column-level average conductance information to construct the ideal reference current during the pre-experiment. Once *CP* is obtained, it is stored and applied to correct the output current in subsequent neural network inference operations. A distinct advantage of the PAAC method is its implementation versatility, allowing for seamless integration into various hardware architectures. In digital-based systems, *CP* is simply applied as a digital multiplication coefficient within the digital logic. Conversely, for fully analog neuromorphic cores, the compensation can be physically realized by adjusting the gain of column-level Transimpedance Amplifiers (TIAs) or by utilizing variable gain amplifiers, as shown in Figure 5a.

Consequently, this method represents a highly efficient one-time calibration process with O(1) computational complexity to effectively mitigate parasitic effects.

To verify the robustness of the linear assumption proposed above, we conducted extensive simulations on randomly generated memristor arrays using HSPICE. In these simulations, we varied three key parameters to cover a wide range of operating conditions—input resistance, line resistance, and array size, with memristor resistances ranging from 10 kΩ to 100 kΩ. For each group of data defined by different line resistance values, input resistance values, and memristor array sizes, 20 pairs of distinct ideal and practical memristor arrays are generated with random voltages (0–0.3 V) and random memristor resistances (10 kΩ to 100 kΩ).

**Figure 6:**
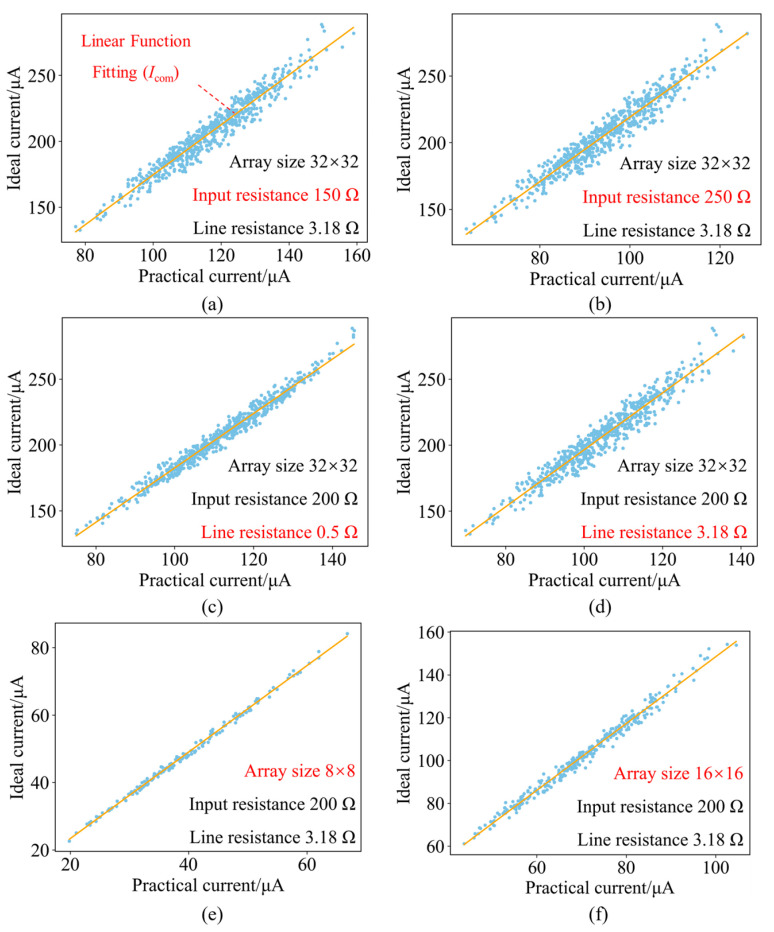
Linear fitting diagrams of output current with variations in array parameters. (**a**,**b**) Linear fitting diagrams of output current with variations in input resistance. (**c**,**d**) Linear fitting diagrams of output current with variations in line resistance. (**e**,**f**) Linear fitting diagrams of output current with variations in the array scale.

Figure 6 illustrates the variations in the fitting function relating the practical output current to the ideal output current as the parasitic resistance and size of the memristor array vary. Figure 6a and Figure 6b show the linear (first-order) function fitting curves of the practical output current versus the ideal output current under input resistances of 150 Ω and 250 Ω, respectively. It can be observed that the increase in input resistance does not affect the correlation between the practical output current and the ideal output current but influences the magnitude of the practical output current, with the horizontal axis coordinates gradually decreasing. This is because the input resistance exerts the same influence on the output current of each column and does not disrupt the correlation relationship between the ideal and practical output currents of each column.

Figure 6c and Figure 6d show the linear (first-order) function fitting curves of the practical output current versus the ideal output current under line resistances of 0.5 Ω and 3.18 Ω, respectively. As line resistance increases, the practical output current becomes more divergent. This is because the output current of columns with larger numbers is more significantly affected by line resistance, resulting in a smaller practical output current, according to Equation (15). This leads to a scenario where the ideal output current of the *j*-th column is lower than that of the *k*-th column, while the practical output current of the *j*-th column is higher than that of the *k*-th column (*j* < *k*). Such cases become more frequent, and the ideal current points scatter more widely as line resistance continues to increase.

Figure 6e,f show that as the array size increases, the number of practical and ideal output currents rises due to the increase in columns, and the magnitude of the practical and ideal output currents increases due to the increase in rows of input voltage. Meanwhile, parasitic resistance has a more severe impact on the output current, resulting in larger errors between the practical output current and the ideal output current. A larger compensation coefficient is needed, according to Equations (14) and (20).

**Figure 7:**
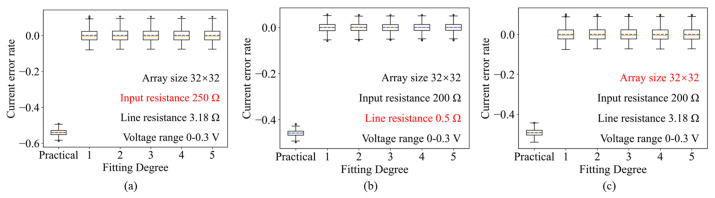
Box plot of the error between the fitted output current and the ideal output current for different array parameters (**a**–**c**).

Furthermore, Figure 7 presents the error analysis (residuals) after applying other compensation. Regardless of variations in parasitic resistance values or array dimensions, the error between the compensated current and the ideal current is significantly minimized. These results confirm that the linear model derived in our theoretical analysis is sufficiently accurate for correcting parasitic distortions, validating the effectiveness of the PAAC method.

To further investigate the relationship between the column-wise compensation factor *CP* and the input pattern, an additional input pattern analysis is conducted. Here, *CP* denotes the proportional factor used to map the practical column output current to the corresponding compensated current. It should be noted that we do not claim that *CP* is strictly constant for every possible input sample. More precisely, the question of interest is whether the *CP* obtained from the fixed-input pre-experiment can serve as a stable, effective, and practically preferable column-wise compensation factor within the realistic input range.

**Figure 8:**
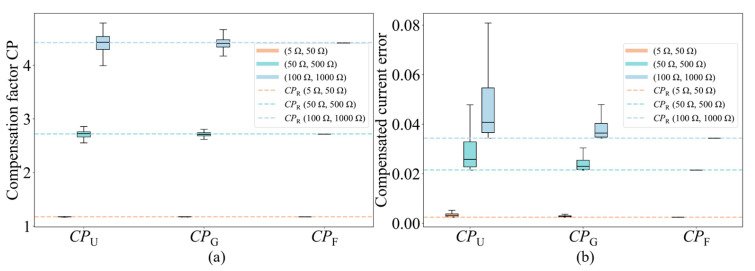
Stability and compensation performance of the column-wise compensation factor *CP* under different input patterns. (**a**) Distribution of compensation factors obtained under three parasitic settings, (5 Ω, 50 Ω), (50 Ω, 500 Ω) and (100 Ω, 1000 Ω), where *CP*_U_, *CP*_G_ and *CP*_F_ denote the compensation factors obtained from uniformly random input, Gaussian random input, and fixed-input pre-calibration, respectively, and the dashed lines represent the reference compensation factor *CP*_R_ obtained from large-sample linear regression. (**b**) Distribution of the compensated current error on the common test set using different compensation factors.

Figure 8 shows the stability and compensation performance of *CP* under different input patterns. In this experiment, a 32 × 32 memristor array network including both input resistance and distributed line resistance is constructed. The memristor resistances are initialized as a fixed random realization uniformly distributed from 10 kΩ to 100 kΩ, and three parasitic settings are considered: (*R*_l_, *R*_in_) = (5 Ω, 50 Ω), (50 Ω, 500 Ω) and (100 Ω, 1000 Ω). For a selected column in the same array realization, 10,000 uniformly random test samples are first generated, and the corresponding practical output current *I*_pra_ and ideal output current *I*_ide_ are calculated from the full resistive network. Four types of compensation factors are then compared: *CP*_U_, *CP*_G_, *CP*_F_, and *CP*_R_, where *CP*_U_, *CP*_G_, and *CP*_F_ are obtained from uniformly random input, Gaussian random input, and fixed-input pre-calibration, respectively, while *CP*_R_ denotes the reference compensation factor obtained from large-sample linear regression.

As shown in Figure 8a, under all three parasitic settings, *CP*_F_ consistently exhibits the smallest dispersion and remains the closest to the regression-based reference *CP*_R._ Figure 8b further shows that the compensated current error obtained using *CP*_F_ is also the closest to that of *CP*_R_. These results indicate that the column-wise compensation factor obtained from the fixed 0.3 V pre-experiment provides not only the simplest implementation, but also the best overall trade-off among stability, compensation accuracy, and practical applicability.

## 4. Experimental Verification

### 4.1. Simulation Verification

We verify the effectiveness of the PAAC method by HSPICE simulation. The parameter setting of array parameters and parasitic resistance in HSPICE is consistent with previous simulation experiments and does not consider the fluctuation of the parasitic resistance and compensates for the output current before calculating the current error.

**Figure 9:**
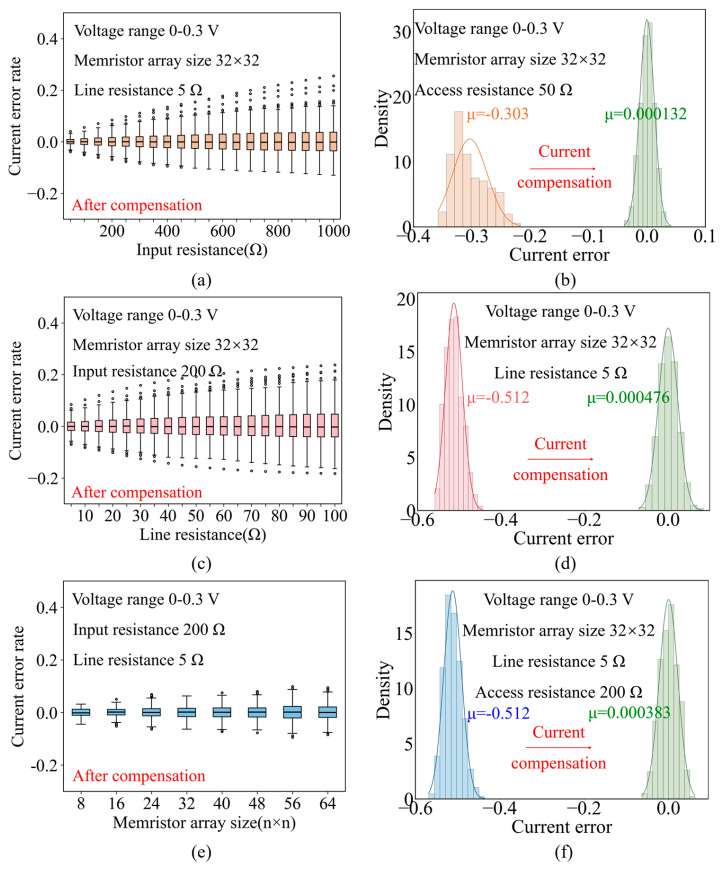
Simulation analysis of the PAAC method. (**a**) The impact of input resistance on the output current after compensation. (**b**) Statistical distribution of current errors due to input resistance before and after compensation. (**c**) The impact of line resistance on the output current after compensation. (**d**) Statistical distribution of current errors due to line resistance before and after compensation. (**e**) The impact of the size of the memristor array on the output current after compensation. (**f**) Statistical distribution of current errors due to the size of the memristor array before and after compensation.

Compensated output current fluctuates more with the increase in the input resistance, but the average error remains approximately 0%, as shown in Figure 9a. Compared with the uncompensated output current in Figure 2b, the output current is significantly optimized, which is close to the ideal output current. When input resistance is 50 Ω, the average error of the output current before and after compensation decreased from −30.3% to −0.013%, as shown in Figure 9b. The result shows that this method can effectively compensate for the input resistance. Similarly, for the line resistance compensation, as shown in Figure 9c,d, the average error of the compensated output current is around 0%. When line resistance is 5 Ω, the average error of the output current before and after compensation decreased from −51.2% to −0.047%. Then the parasitic resistances are fixed with different sizes of arrays. As shown in Figure 9e,f, the average output current error is around 0% and when the size of array is 32 × 32, the average error of the output current before and after compensation decreased from −51.2% to −0.038%. The simulation experiment results show that the PAAC method can greatly reduce the influence of parasitic resistance in different sizes of memristor arrays, restoring the output current distortion.

### 4.2. Application Verification

Feasibility verification on board-level test system

**Figure 10:**
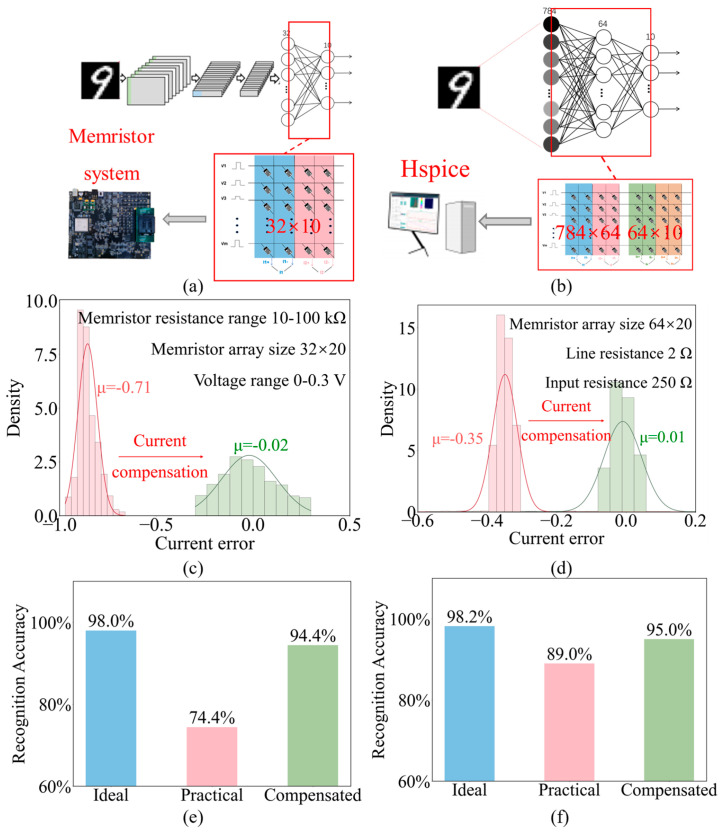
MNIST handwritten digits classification analysis of the PAAC method. (**a**) Schematic diagram of the Convolutional Neural Network in a board-level test system. (**b**) Schematic diagram of the Multilayer Perceptron neural network in HSPICE. (**c**) Statistical distribution of current errors before and after compensation in the board-level test system. (**d**) Statistical distribution of current errors before and after compensation in HSPICE. (**e**) Classification performance of neural networks implemented by the ideal current, the practical current and the compensated current in a board-level test system. (**f**) Classification performance of neural networks implemented by the ideal current, the practical current and the compensated current in HSPICE.

To first verify the feasibility and practicality of the PAAC method in a real-world hardware environment, we implemented the MNIST handwritten digit classification on a board-level test system containing a memristor array chip. As shown in Figure 10a, the system includes weight read/write circuits, input generation circuits, and output readout circuits to support network weight programming and inference operations.

Due to the limited scale of the specific memristor chip used in this system (1 k, 32 × 32 array), we adopted a specific mapping strategy. A Convolutional Neural Network (CNN) was trained offline, and its final output layer (fully connected) was mapped onto the 32 × 20 region of the memristor array, while the preceding layers were implemented in Python 3.10. The board-level test system adopts a modulation strategy with variable gate voltage to precisely program memristor weights [23]. The practical output currents were collected from the Source Lines (SLs) for discrimination.

The experimental results demonstrate the significant impact of real-world parasitic effects and the efficacy of the compensation. As shown in Figure 10c,e, the average output current error before compensation was as high as −71%, heavily degrading the classification accuracy to 74.4%. After applying the PAAC method, the average current error was drastically reduced to −2%, and the classification accuracy recovered to 94.4%. These hardware-based results confirm that the PAAC method effectively mitigates parasitic distortions in practical scenarios with complex environmental noise and interconnects.

It should be noted that the above board-level validation is based on a hybrid software–hardware implementation, in which the preceding CNN layers are executed in software while only the final fully connected layer is mapped to the memristor array hardware. This is a practical compromise imposed by the current prototype scale, and its main purpose is to verify the feasibility of the PAAC method in a realistic hardware signal chain rather than to emulate a complete end-to-end hardware neural network system.

Under such a configuration, the absolute board-level error is affected not only by the parasitic resistance inside the array, but also by additional factors such as quantization and scaling mismatch, DAC-driving deviation, readout gain/offset error, noise, and software–hardware interface imperfections. Therefore, a residual gap between the compensated board-level result and the ideal result is expected.

However, the key objective of the board-level experiment is not the absolute error of the hybrid implementation itself, but the relative improvement before and after compensation under the same software front-end, interface path, hardware array, and readout condition. Since these conditions are kept unchanged in the before/after comparison, the interface-related errors are shared by both cases and do not alter the main conclusion regarding the effectiveness of the PAAC method. Under these identical conditions, the average current error is reduced from about −71% to about −2%, while the classification accuracy is improved from 74.4% to 94.4%, demonstrating that PAAC provides substantial correction for the dominant parasitic distortion in a realistic hardware loop.

Scalability Verification via Large-scale HSPICE Simulation

While the hardware experiments confirmed the method’s feasibility, the limited array size (32 × 32) of the prototype chip does not fully reflect the severity of parasitic effects in larger-scale integration, where line resistance accumulates significantly. To further verify the scalability of the PAAC method on larger networks, we constructed a full Multilayer Perceptron (MLP) neural network in HSPICE.

As shown in Figure 10b, the MLP consists of three layers: an input layer (784 neurons), a hidden layer (64 neurons), and an output layer (10 neurons). This network was fully mapped onto two large-scale memristor crossbar arrays, utilizing a resistance range of 10 kΩ to 100 kΩ and an input pulse range of 0–0.3 V. The network was pre-trained using Python to achieve a theoretical accuracy of 98.2%.

In the simulation, the vector–matrix multiplication was performed according to Ohm’s law and Kirchhoff’s laws, incorporating the distributed parasitic resistance models. As shown in Figure 10d, due to the voltage division caused by parasitic resistances in these larger arrays, the uncompensated practical output current exhibited a significant average error of −35%. Subsequently, the PAAC method was applied to correct the currents. The statistical analysis shows that the average error was reduced to −1%, demonstrating that the linear compensation model remains highly accurate even in larger arrays. Consequently, as shown in Figure 10f, the classification accuracy of the neural network improved from 89% (uncompensated) to 95% (compensated), approaching the ideal accuracy of 98.2%.

These simulation results, combined with the hardware validation, confirm that the PAAC method is not only practical for real devices but also scalable to larger neuromorphic systems.

## 5. Discussion

Although PAAC significantly reduces the dominant distortion caused by parasitic resistance, a residual gap still remains between the compensated board-level result and the ideal result. This indicates that, besides the dominant parasitic-resistance-induced distortion, other error sources still exist in the practical system. These residual errors mainly come from device nonidealities, programming errors, readout-chain and interface errors, as well as higher-order nonlinear effects that are not explicitly included in the present linear approximation model. Therefore, further reducing this gap will require future extensions such as interface-aware calibration, device-variability-aware compensation, and higher-order correction terms.

The present work uses MNIST mainly to clearly isolate the effect of parasitic resistance compensation on inference accuracy under the current prototype size and mapping capability. Although this setting is appropriate for demonstrating feasibility, further validation on more complex datasets such as CIFAR-10 is also of great importance. In addition, the current board-level prototype only supports the hardware mapping of the final fully connected layer. Therefore, the parasitic effects on convolution-heavy layers have not yet been experimentally validated at the hardware level. Extending the PAAC method to more complex datasets, deeper network mappings, and convolution-intensive layers remains an important future direction.

Another point worth emphasizing is that column-wise compensation is not the only possible compensation form. In certain architectures, row-wise or global compensation may also be meaningful. However, in the crossbar-computing scheme considered in this work, the final inference result is read out in the form of column currents, and therefore the parasitic distortion is most directly reflected at the column-output level. Based on this, column-wise compensation in the PAAC method can directly correct the final inference quantity. In contrast, although global compensation is simpler to implement, it is usually less accurate because it ignores inter-column parasitic variation, while row-wise or block-wise compensation may become valuable extensions in other architectures or larger arrays.

It should also be noted that, when input resistance and line resistance coexist, higher-order coupling terms may still exist, and a single linear factor may not fully reconstruct all local node behaviors. However, the purpose of the PAAC method is not to recover the entire internal circuit state with node-level precision, but to correct the dominant distortion of the column output current with as low complexity as possible. The experimental and simulation results show that this column-wise linear compensation is already effective in recovering inference accuracy. In future work, block-wise compensation, intercept-including models, and higher-order nonlinear correction may be explored to more fully account for the coupling among parasitic effects.

Finally, the current PAAC method focuses on parasitic resistance compensation during the inference stage, where the programmed memristor states are assumed to remain unchanged during one computation. Therefore, the present model does not explicitly address weight update dynamics or device plasticity during online training. In future work, the PAAC method may be extended through recalibration after weight updates, periodic pre-calibration, or integration with write-error-aware models, thereby making it compatible with more general training–inference integrated memristor systems. More broadly, the underlying PAAC method may also be extended to other complex parasitic scenarios, such as parasitic capacitance and inductance.

## 6. Conclusions

In summary, this work systematically analyzed the effect of parasitic resistance in memristor-based neuromorphic computing and proposed the PAAC method based on linear approximation models. As summarized in Table 1, compared with existing compensation approaches, the proposed PAAC method mitigates the dominant parasitic-resistance-induced distortion without requiring prior extraction of the exact parasitic resistance values, while maintaining very low computational overhead. In the present implementation, only four multiplication operations are required to calculate the compensation factor, making the method suitable for low-complexity hardware deployment.

The effectiveness of the PAAC method was validated through a comprehensive two-stage evaluation. First, board-level hardware verification demonstrated the practical feasibility of the method in a realistic hardware signal chain, reducing the average current error from 71% to 2% and improving the classification accuracy from 74.4% to 94.4%. Second, large-scale HSPICE simulations verified its scalability, showing that the proposed method can still effectively reduce current error from 35% to 1% and restore neural network performance from 89% to 95% in larger arrays.

Overall, the PAAC method proposed in this work provides a simple, robust, and practically feasible solution for compensating dominant parasitic-resistance-induced distortion in memristor-based neuromorphic computing, thereby facilitating the implementation of large-scale memristor-based neuromorphic hardware systems.

## Figures and Tables

**Table 1:** Comparison with the previous work of the compensation in memristor array.

	Parasitic Resistance Effect	Time Complexity	Platform for Verification	Averaged Output Error		Accuracy of Neural Networks		
				Before	After	Array Size	Before	After
X. Zhu [16]	Line resistance	O(n)	Software	20%	1.83%	400 × 50 × 10	80.26% (MNIST)	94.78%
T. V. Nguyen [17]	Both line resistance and input resistance	O(n)	Software	65.2%	8.60%	784 × 200 × 10	90.4% (MNIST)	95.1%
N. Lepri [18]	Line resistance	O(n)	Software	None	None	784 × 64 × 10	59% (MNIST)	96.6%
P. Tong [19]	Input resistance	O(1) 14 multiplications and 5 additions.	Hardware	60%	12%	32 × 4 (Fully Connected Layer)	89.8% (EEG-signals)	91.20%
This work	Both line resistance and input resistance	O(1) 4 multiplications	Software	35%	1%	784 × 64 × 10	89% (MNIST)	95%
			Hardware	71%	2%	32 × 10 (Fully Connected Layer)	74.4% (MNIST)	94.4%

## Data Availability

The data presented in this study are available on request from the corresponding author.

## Abbreviations

The following abbreviations are used in this manuscript:

PAAC	Parameter-Agnostic Adaptive Compensation
IR-drop	Voltage drop caused by parasitic resistance in the current path
VMM	Vector–Matrix Multiplication
BL	Bit Line
SL	Source Line
WL	Word Line
TEM	Transmission Electron Microscopy
TIA	Transimpedance Amplifier
CNN	Convolutional Neural Network
MLP	Multilayer Perceptron
CMOS	Complementary Metal-Oxide-Semiconductor
